# Perception and recognition of faces in adolescence

**DOI:** 10.1038/srep33497

**Published:** 2016-09-20

**Authors:** D. Fuhrmann, L. J. Knoll, A. L. Sakhardande, M. Speekenbrink, K. C. Kadosh, S. -J. Blakemore

**Affiliations:** 1 Institute of Cognitive Neuroscience, University College London, London, United Kingdom; 2 Department of Experimental Psychology, University College London, London, United Kingdom; 3 Department of Experimental Psychology, University of Oxford, Oxford, United Kingdom; 4 School of Psychology, University of Surrey, Guildford, United Kingdom

## Abstract

Most studies on the development of face cognition abilities have focussed on childhood, with early maturation accounts contending that face cognition abilities are mature by 3–5 years. Late maturation accounts, in contrast, propose that some aspects of face cognition are not mature until at least 10 years. Here, we measured face memory and face perception, two core face cognition abilities, in 661 participants (397 females) in four age groups (younger adolescents (11.27–13.38 years); mid-adolescents (13.39–15.89 years); older adolescents (15.90–18.00 years); and adults (18.01–33.15 years)) while controlling for differences in general cognitive ability. We showed that both face cognition abilities mature relatively late, at around 16 years, with a female advantage in face memory, but not in face perception, both in adolescence and adulthood. Late maturation in the face perception task was driven mainly by protracted development in identity perception, while gaze perception abilities were already comparatively mature in early adolescence. These improvements in the ability to memorize, recognize and perceive faces during adolescence may be related to increasing exploratory behaviour and exposure to novel faces during this period of life.

Faces are of unique importance in everyday life. Recognition of faces is fundamental to building and maintaining relationships^1^. Faces also provide social signals such as information about other people’s mental states and emotions^2^ and facilitate communication and social learning^3^. Models of face cognition mainly distinguish between two sub-components: *face memory*, the ability to learn and recognize known faces, and *face perception*, the ability to discriminate facial features and configurations^4^. Face memory and face perception are face-specific skills that are distinct from other abilities such as object cognition^5^.

The ability to recognize people from their faces has its origins early in development^6^, but face memory expertise follows a protracted course of development thereafter. Face memory has been shown to improve rapidly between 6 and 10 years and then levels off, or even dips with the onset of puberty, and then rises again later during the teenage years^7^. Other studies have shown linear improvements between childhood and adulthood^8^,^9^. There are gender differences in face memory. Adult females generally perform better than males in face memory^10^,^11^,^12^ and some studies have found a female advantage in face memory across the adolescent age range as well^9^. There is disagreement as to when face memory matures. Proponents of the *late maturation account* argue that face memory does not mature until at least 10 years of age and is likely to be driven by experience of faces - by one’s ‘face diet’^13^. Proponents of the *early maturation account*, in contrast, contend that experience has little effect on face memory development and that quantitative improvements in face memory after 3–5 years are due to improvements in general cognitive ability^14^.

Similarly, for face perception, early maturation accounts compete with late maturation accounts^15^,^16^. The disagreement may in part be attributable to the fact that the perception of different face aspects such as identity, expression or gaze, develops at different rates^17^. These face aspects are processed in different ways by the face-perception network of the brain^18^,^19^, and gaze perception generally matures earlier than identity or expression perception, with identity perception maturing last^17^,^18^.

This developmental pattern may be linked to different rates of maturation of the underlying cognitive processing strategies that are employed for different face aspects. Two main cognitive strategies have been identified: configural and featural processing^15^. Configural processing refers to the processing of the overall layout of the face while featural processing describes the processing of a face via its features in isolation. Previous studies have shown that configurations of faces are key to recognizing facial identities^20^, while featural strategies are more often used to determine the direction of gaze^17^ and a mix of both is used to perceive expressions^21^. Featural face processing allows the perceiver to focus on one specific facial feature and has been shown to be proficient from a very young age^15^. In contrast, configural face processing is more complex, as it relies on the integration of several facial features. It is a cognitive strategy that requires much training and which extends well into the second decade of life^15^.

Only a relatively small number of studies have investigated the development of face aspect-specific perception in childhood and adulthood. Children tend to be slower than adults at detecting changes in identity and expression, even though face perception abilities on the whole are adult-like by 7 years^17^. Mirroring these behavioural changes between childhood and adulthood, the face perception network, including areas such as the fusiform face area and inferior occipital gyrus (IOG), undergo protracted changes in adolescence^18^. In a previous study, we showed that children, adolescents and adults differed in the adaptation of the IOG to different face aspects^18^. Children focussed on gaze even when they were instructed to detect changes in identity. In contrast, adults’ IOG activation was more strategy-driven. Adolescents’ IOG was not clearly adapted to different face aspects, with activation being more heterogeneous, possibly reflecting a shift in processing strategies with age. When such a shift occurs, and when in adolescence the ability to process changes in identity and expression become adult-like, remains unclear.

In the current study, we investigated face cognition in 661 adolescents and adults aged 11–33 (397 females). Participants were split into four age groups: younger adolescents (11.27–13.38 years); mid-adolescents (13.39–15.89 years); older adolescents (15.90–18.00 years); and adults (18.01–33.15 years). We investigated age-related changes in the two core face cognition abilities, face perception and face memory, between adolescence and adulthood. Participants completed the Cambridge Face Memory Test^22^, which measures the ability to memorise and recognise faces, and the Face Same-Different face perception task^17^, which measures the ability to recognise changes in identity, expression or gaze between faces. The latter task is designed to prevent participants from using a simple strategy, such as always focussing on the eyes by changing only one of these face aspect at a time, and never a mix of aspects.

We predicted that face cognition abilities would improve from early adolescence to adulthood and investigated whether these developmental patterns differed between face memory and perception. We also examined gender differences in face cognition, and predicted that females would outperform males. Finally, we explored developmental differences in the perception of different face aspects (identity, expression and gaze). We predicted that the ability to perceive changes in gaze would mature earlier than the ability to perceive changes in identity.

## Results

### The development of face memory and face perception

To assess whether developmental trajectories differed between face memory and face perception, we pooled the data for both tasks, generating an overall index of face cognition ability. We then investigated age effects in overall face cognition before determining whether these age effects where moderated by task (face perception versus face memory). We controlled for age group differences in general cognitive ability in all analyses (see Table 1 and Supplementary Results in Supplementary Information).

**Figure 1:**
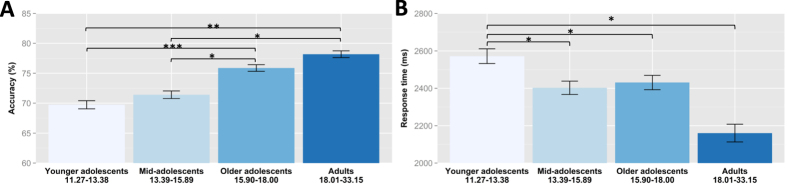
Face cognition performance (panel A: accuracy, panel B: response times) in four age groups: younger adolescents (11.27–13.38), mid-adolescents (13.39–15.89), older adolescents (15.90–18.00) and adults (18.01–33.15). Results are showen averaged over the face prcoessing and face memory task with standard error bars. *p < 0.05, **p < 0.01, ***p < 0.001 (Bonferroni-corrected for multiple-comparison).

There were significant differences in overall face cognition accuracy between age groups (*χ*^2^(3) = 24.40, *p* < 0.001). Younger and mid-adolescents performed significantly worse than the two older age groups in face cognition (Fig. 1A, Supplementary Table S2). There were also significant differences in response times on correct trials between age groups (*χ*^2^(3) = 18.46, *p* < 0. 001). Response times were significantly slower in younger adolescents than in all older age groups. However, the contrast between mid-adolescents and the older age groups did not reach significance for response times (Fig. 1B, Supplementary Table S3). The age effects did not differ between tasks (accuracy: *χ*^2^ (3) = 4.47, *ns*; response times: *χ*^2^(3) = 4.41, *ns*) indicating that face memory and perception followed similar developmental trajectories.

### Gender differences in face memory and face perception

There were no differences in reasoning scores between genders (*F*(1,654) = 2.52, *ns*) indicating that general cognitive ability was matched. There was a main effect of gender on overall face cognition accuracy (*χ*^2^(1) = 13.48, *p* < 0.001) but not response times (*χ*^2^(1) = 0.17, *ns*). For both dependent measures, there was an interaction between gender and task (accuracy: χ^2^(1) = 8.00, p = 0.005; response times: *χ*^2^(1) = 9.01, *p* = 0.003) indicating that the effect of gender differed between face memory and face perception. For accuracy, females outperformed males in face memory but not face perception. Response times followed the same pattern (Table 2). Developmental trajectories did not differ between genders (accuracy: *χ*^2^(3) = 1.10, *ns*; response times: *χ*^2^(3) = 2.47, *ns*).

### Development of face aspect perception

The face perception task measured the ability to correctly detect changes in three face aspects: identity, expression and gaze. We investigated whether there were differences in the ability to process these face aspects and whether the developmental trajectories for identity, expression and gaze perception differed. There was a main effect of face aspect on accuracy (*χ*^2^(2) = 313.96, *p* < 0.001). Participants performed significantly better in gaze perception (Mean_gaze_ = 0.72, SD_gaze_ = 0.17) than in identity perception (Mean_identity_ = 0.55, SD_identity_ = 0.22, *z* = 16.59, *p* < 0.001) or expression perception (Mean_expression_ = 0.58, SD_expression_ = 0.20, *z* = 14.32, *p* < 0.001). The difference between identity and expression perception did not survive correction for multiple comparisons (*z* = 2.39, *p* = 0.051). The face aspect also affected response times (*χ*^2^(2) = 73.44, *p* < 0.001). Following the same pattern as accuracy, participants responded significantly faster in gaze perception (Mean_gaze_ = 1596.92, SD_gaze_ = 351.95) than in identity perception (Mean_identity_ = 1761.81, SD_identity_ = 483.19, *t* = −8.55, *p* < 0.001) or expression perception (Mean_expression_ = 1691.43, SD_expression_ = 428.05, *t* = −4.75, *p* < 0.001). They were also quicker in expression than identity perception (*t* = −3.08, *p* < 0.001).

**Figure 2:**
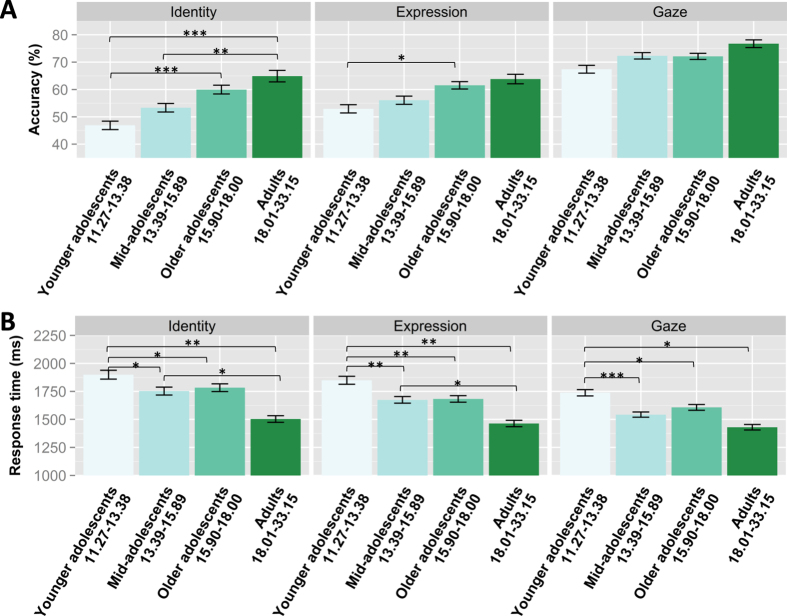
Face perception performance (panel A: accuracy, panel B: response times) by face aspect in four age groups: younger adolescents (11.27–13.38), mid-adolescents (13.39–15.89), older adolescents (15.90–18.00) and adults (18.01–33.15). Results are shown for identity, expression and gaze perception with standard error bars. *p < 0.05, **p < 0.01, ***p < 0.001 (Bonferroni-corrected for multiple-comparison).

The effect of face aspect was moderated by age group for accuracy (*χ*^2^(6) = 14.84, *p* = 0.022), indicating that the ability to correctly identify changes in identity, expression or gaze differed between age groups. There were developmental differences in identity perception such that younger adolescents were less accurate than older adolescents and adults and mid-adolescents were less accurate than adults. Younger adolescents were also worse than older adolescents in expression perception while there were no developmental differences in gaze perception (Fig. 2A, Supplementary Table S4). To assess whether the developmental differences in identity and expression perception were significantly greater than in gaze perception we inspected contrasts. The difference between younger adolescents and the older age groups was significantly greater in identity than in gaze perception (*z* = −2.23, *p* = 0.026), the difference between mid-adolescents and the older age groups was also significantly greater in identity than gaze perception (*z* = −2.58, *p* = 0.010, SI). All other comparisons, including all comparisons between gaze and expression perception were not significant (p > 0.05; Supplementary Table S5), indicating that developmental effects were mainly restricted to identity perception. The effect of face aspect was not moderated by age group for response times (*χ*^2^(6) = 7.58, ns). There were developmental differences in all face aspects. Younger adolescents were significantly slower than all older age groups for all three face aspects, and mid-adolescents were significantly slower than adults in identity and expression perception (Fig. 2B, Supplementary Table S6). The developmental differences between younger adolescents and the older age groups were not stronger in identity or expression perception than gaze perception (Supplementary Table S7).

In summary, there were improvements with age in identity perception, both in accuracy and speed, while expression and gaze perception improved in speed only.

## Discussion

The results from this large scale, cross-sectional study demonstrate that face cognition undergoes protracted development: younger adolescents’ and mid-adolescents’ face memory and face perception abilities were less proficient than those of older adolescents and adults. Most studies on the development of face cognition abilities have focussed on early and mid-childhood, with many studies suggesting that face cognition abilities are mature by 3–5 years of age^14^. However, other studies have shown improvements from early adolescence to adulthood, a finding that we replicated here^8^,^9^,^23^. Our study showed that the main period of face cognition development in adolescence is roughly between 11 and 16 years. This was the case for both face memory and face perception, the two core components of face cognition. General cognitive ability (as measured by matrix reasoning) predicted face cognition scores but showed a different developmental trajectory with continuous improvements throughout adolescence and into adulthood. Our results therefore indicate that the development of face cognition in adolescence does not support the general cognitive ability explanation of improvements in face cognition beyond childhood^14^. As the current study assessed within-category differentiation of different faces aspects, future studies will need to ascertain the domain-specificity of these results by providing comparable memory and perception tests with non-face objects.

The effects of gender differed between the two sub-components of face cognition, with a female advantage for face memory but not for face perception. This pattern did not change with age. Previous studies have shown a female advantage in face memory in adults^10^,^11^,^12^ and in adolescents^9^, which we replicated in the current study. Some studies have also found a female face *perception* advantage in adults, which was not replicated here^10^. The female advantage in face cognition is thought to be driven by female participants scanning face stimuli more than males^11^. One explanation for why we found gender differences in face memory but not in face perception is that the increased face scanning by females may lead to gender differences in tasks in which face stimuli are presented for a long period of time (up to 20 s in the case for the face memory task) and preclude gender differences in tasks where the stimuli are only presented for a short time (500 ms for the face perception task). Gender differences in face cognition in adults are partly explained by greater social interest and involvement in females than males^10^, but this remains to be tested in adolescents.

An inspection of the ability to recognise changes in the three face aspects manipulated in the face perception task – identity, expression and gaze – revealed that developmental effects in the face perception task were driven mainly by differences in identity perception between age groups. Younger adolescents and mid-adolescents were less accurate than older adolescents and adults in identity perception, but not in gaze perception. This supports the late maturation account of face cognition by showing not only quantitative differences between adolescent age groups but also qualitative differences, with identity perception maturing only in mid-adolescence. This pattern also matches models of early maturation of featural versus late maturation of configural perception^15^,^17^. Perceiving identity changes requires mainly configural perception, whereas perceiving a change in gaze direction recruits purely featural perception^17^,^18^.

Mid-adolescence may be of particular importance not only for the development of face cognition but also for the development of social cognition in general. Developmental models^24^ and empirical evidence^25^ indicate a perturbation of face cognition with the onset of puberty. The ensuing period of rapid cognitive and neurological development may provide an ideal substrate for learning^26^,^27^. Exploratory behaviour in adolescence^28^ may lead to more exposure to novel faces than earlier in life and new social roles in adolescence may increase the focus on facial information such as attractiveness and status^24^. This may then provide the environmental enrichment necessary for becoming a face expert. This interpretation fits with the perceptual expertise account of face perception^29^. Extensive experience with a specific category of objects, not just faces, is thought to lead to more efficient mental representations, perhaps through “holistic” encoding. The concept of holistic encoding is similar to configural perception and describes the ability to process a stimulus as a whole rather than the sum of its parts^30^.

In conclusion, face memory and face perception abilities mature relatively late in development, between early and late adolescence. Improvements in face perception over adolescence were driven by increased identity perception abilities. These improvements over adolescence may be related to an increased exposure to novel faces during this period of life.

## Methods

### Participants

We recruited 821 participants from 16 local schools in the London area (adolescents) and through the UCL participant pools and posters (adults) for a training study^38^ of which data at baseline was analysed here. Of this sample, 661 participants were included in the current analysis (mean age at baseline = 16.21 years, SD = 4.12, age range = 11.27–33.15 years, 397 females). Exclusion criteria were: missing baseline data (N = 1); missing parental consent for adolescents (N = 123); report of developmental conditions including ADHD and dyslexia (N = 34). Adolescents were split into three age groups of equal size and adults were included as a fourth age group (Table 1). The study was carried out in accordance with UCL Research Ethics Guidelines and was approved by the UCL Research Ethics Committee. Informed consent was obtained from all participants included in this study.

### Design

A 4 × 2 × 2 design with age group (younger adolescents, mid-adolescents, older adolescents, adults) and gender as between-subjects factors and task (face perception, face memory) as a within-subjects factor was employed. For the face perception task, face aspect was investigated as an additional within-subjects factor with 3 levels (identity, expression, gaze).

### Testing procedure

The testing procedure was previously described in^38^ participants were tested on a battery of tasks including two face cognition tasks: a face perception and a face memory task (see below for details of these tasks). Testing was carried out using an online platform developed by the research team and a software company (www.cauldron.sc).

Participants completed the testing session in groups of 3–48 in school (adolescents) or in a University computer room (adults), using laptops, tablets or desktop computers. Responses were made using a mouse or touchscreen. The order of the tasks was counterbalanced between testing groups.

An experimenter gave instructions before the task. Participants then completed practice trials until three were completed correctly. For the face memory task, 22 participants completed more than 3 practice trials. On average, these participants needed 5.2 practice trials to proceed to the task and never more than 7. For the face perception task, 173 participants completed more than 3 practice trials. On average, these participants needed 4.4 practice trials to proceed to the task and never more than 8. All participants completed three practice trials successfully during the testing session and proceed to the task. Participants were given visual feedback on their performance in the practice trials only.

### Face memory task

**Figure 3:**
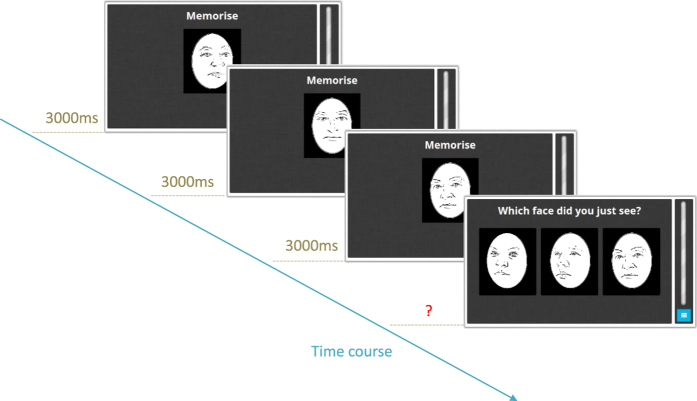
Procedure for block 1 of the face memory task^22^. A face was presented from three angles for 3000 ms each, followed by a blank screen for 500 ms after each angle (not shown). A panel of three faces was then presented including the target face just studied and two distractors. Participants chose one face. After providing a response, the next trial started immediately. This AFC trial was repeated twice more. In the second block, the same 6 target faces were presented simultaneously for 20 s, and this was followed by 18 AFC trials. In the third block, the same 6 target faces were presented simultaneously for 20 s, but a Gaussian noise mask (50% noise) was added to the faces in the 18 AFC trials that followed. There was no time restriction for the response in any of the blocks.

An adaptation of the Cambridge face memory task (CFMT) was used to assess the ability to learn and recognise unknown faces using a 3-Alternative-Forced Choice (3-AFC) test^22^. Participants were asked to memorise target faces and then find a target face in a panel of three faces. There was only ever one target face in the panel of three, the other two were distractor faces that had not been memorised (Fig. 3).

The task took 9 min or 54 trials to complete, whichever came first. The task was shortened from the original 72 trials^22^ to due to time restrictions in schools. The three blocks of the original CFMT were preserved but block 2 and 3 were shortened to match the number of trials in block 1. Adults’ accuracy in our adaptation of the CFMT was 82.02% (SD: 7.63) and similar to adults’ performance in the original CFMT, in which accuracy was 80.4% (SD: 11.0). The third block was repeated if participants finished all trials before the time limit but data from these repeated trials was not included in the analysis.

A set of 126 face stimuli matching the specifications of the original CFMT was created for the purpose of the present study and the larger training study, comprising three testing sessions in total. Photographs of 42 Caucasian males from three angles (frontal/left quarter profile/right quarter profile) were obtained from the Facial Recognition Technology (FERET) database^31^. Black and white images were cropped to exclude external features of the face (hair etc.) using GIMP. The size of each face was standardized to 180 × 245 pixels and luminance of the image was set to a value of 110 using GIMP’s Levels function.

### Face perception task

**Figure 4:**
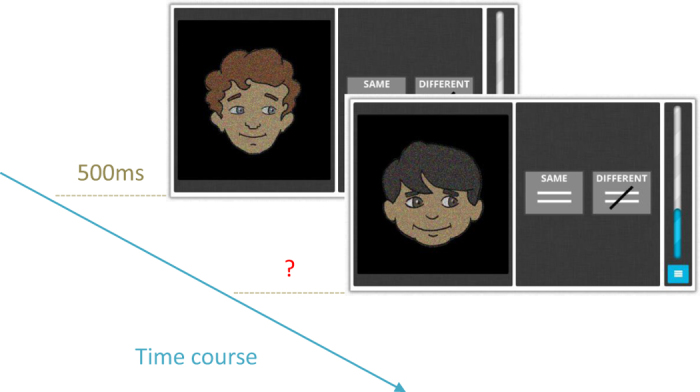
Procedure for the face perception task^17^. Screen shots show stimuli from practice trials. Each trial started with a fixation cross presented for 800 ms, followed by the first face presented for 500 ms, and then another fixation cross for 800 ms, and then the second face until a response was logged or 5000 ms passed, whichever came first. The two possible response options (same/different) were shown simultaneously with the presentation of the two faces. The next trial started immediately after the participant had entered their response. The trial displayed shows an identity change.

The face perception task measured the ability to process featural and configural changes in faces^17^. Participants were asked to decide whether two consecutively presented faces presented were the same or different (Fig. 4). Faces were considered to be different with regard to changes in any of the following face aspects: gaze direction (left/right), expression (happy/sad) or identity (person A/person B). Participants were informed that faces should be classified as the same only if they are exactly the same across all three face properties.

A testing session took 7.5 min or 48 trials to complete, whichever came first. 16 out for 658 participants completed fewer than 48 trials. They still completed 89.7% of trials on average and never less than 62.5%. If participants finished the 48 trials within the 7.5 min time limit, the set of faces was presented again, but the data were not included in the analysis. In half the trials, faces were the same, in the other half; faces differed (24 trials). In the trials in which faces differed, a third showed changes in expression, a third in identity and a third in gaze (8 trials per aspect). Trial difficulty was varied by adding noise masks of increasing strengths (25–81% in 8% steps), except the first two trials, which had a noise mask of 25%.

Photos of two female, Caucasian faces were taken under standardised lighting conditions for the purpose of this experiment. Four photos were obtained for each face: happy expression-gaze left, happy expression-gaze right, sad expression-gaze left, and sad expression-gaze right. Using the picture editing software GIMP, coloured photos were scaled to a uniform size and cropped to exclude external features of the face (hair etc.) Using GIMP’s Levels function, the lightness of the image and mean RGB values were standardized (Luminance: 105, R: 105, G: 75, B: 70). Task difficulty was increased by presenting the images with a Gaussian noise mask of varying strength (25%, 33%, 41%, 49%, 57%, 65%, 73% or 81% noise).

### Statistical analysis

General and Generalized linear mixed-effects models were implemented in *R*^32^, *lme4*^33^ and *lmerTest*^34^ to assess differences in task performance between age groups. Trials with a response time under 250 ms were excluded from the analysis. To asses age group differences in face cognition, a logistic model predicting correct/incorrect responses (accuracy) and a general linear model predicting response times in correct trials were computed. Helmert-coded fixed effects included were age group, task and gender as well as all possible 2-way interactions and the 3-way interaction. Z-scored performance in the relational reasoning task for each participant was included as covariate to control for differences in general cognitive ability between age groups (see Table 1 and Supplementary Results in Supplementary Information). Nested random intercepts for participant ID and school/university were used to reflect the repeated-measure design and clustered nature of participants tested. Planned comparisons of age group differences were carried out using *lsmeans*^35^ and Bonferroni-adjusted for multiple comparisons. To investigate the effect of face aspect on performance in the face perception task, two separate models predicting accuracy and response times in correct trials were computed. Age group and face aspect as well as their interaction were included as fixed effects. The covariate and random effects were computed as described above. Custom contrasts were computed using package *multcomp*^36^ to investigate differences in performance dependent on face aspect and comparing differences between age groups within face aspects. These contrasts were Bonferroni-corrected for multiple comparisons. Finally, one model for accuracy and one for response times in face perception were computed, which were identical to the models described above except for the fact that face aspect was dummy-coded with gaze perception as the reference group. This allowed inspection of contrasts of the interaction of face aspect with age group using the *summary* function. See Supplementary Table S8 for estimates of effect sizes for the models used.

## Additional Information

**How to cite this article**: Fuhrmann, D. *et al.* Perception and recognition of faces in adolescence. *Sci. Rep.* **6**, 33497; doi: 10.1038/srep33497 (2016).

## Supplementary Material

Supplementary Information

## Figures and Tables

**Table 1:** Demographic information including age range, number of participants for each gender and relational reasoning accuracy (Mean_RR_) and standard deviation (SD_RR_).

Age group	Age range	Gender	N	Mean_RR_	SD_RR_
Younger adolescents	11.27–13.38	female	118	0.63	0.16
		male	67	0.57	0.18
Mid-adolescents	13.39–15.89	female	89	0.68	0.15
		male	96	0.68	0.17
Older adolescents	15.90–18.00	female	109	0.73	0.13
		male	77	0.72	0.15
Adults	18.01–33.15	female	81	0.81	0.12
		male	24	0.79	0.17

The relational reasoning task was based on Raven’s matrices, a standard measure of IQ and general cognitive ability^37^,^38^.

**Table 2:** Mean accuracy and response times (standard deviation in brackets) in the face memory task and face perception task for females and males as well as test statistics comparing genders.

		Female	Male	Comparison
Accuracy	Face memory	0.79 (0.11)	0.75 (0.13)	*z* = 4.54, *p* < 0.001
	Face perception	0.70 (0.09)	0.68 (0.09)	*z* = 1.83, *ns*
Response times (ms)	Face memory	3144.53 (880.56)	3337.60 (935.46)	*t* = −2.29, *p* = 0.022
	Face perception	1626.21 (294.67)	1585.31 (299.01)	*t* = 1.67, *ns*
