# Supplementary material for: Perception and recognition of faces in adolescence
Source: Sci Rep. 2016 Sep 20;6:33497. doi: 10.1038/srep33497 (PMC5028728; doi:10.1038/srep33497)
Supplement: Supplementary Information [file srep33497-s1.pdf]

Supplementary Information

Perception and recognition of faces in adolescence

D. Fuhrmann[1], L.J. Knoll[1], A.L. Sakhardande[1], M. Speekenbrink[2], K. Cohen Kadosh[3,4] & S-J Blakemore[1]*

* Corresponding author, email: delia.fuhrmann.13@ucl.ac.uk

[1] Institute of Cognitive Neuroscience, University College London, London, United Kingdom
[2] Department of Experimental Psychology, University College London, London, United Kingdom
[3] Department of Experimental Psychology, University of Oxford, Oxford, United Kingdom
[4] School of Psychology, University of Surrey, Guildford, United Kingdom

Supplementary Results

Age group differences in general cognitive ability

General cognitive ability differed between age groups as indexed by accuracy in a non-verbal matrix reasoning task similar to those included in IQ tests (see [37,38] for details of the task). Reasoning scores increased with age (Table 1). There were significant differences at $p < 0.05$ between all age groups ($F(3,654) = 42.28$, $p < .001$; see Supplementary Table S1). Reasoning scores were therefore included as a covariate in all analyses to control for age-group differences in general cognitive ability (see Methods section).

Supplementary Table S1. Planned contrasts comparing relational reasoning accuracy overall between age groups. Abbreviations: YA – younger adolescents, MA – mid-adolescents, OA – older adolescents, Ad – adults, * $p < 0.05$, ** $p < 0.01$, *** $p < .001$

Contrast	Estimate	SE	df	*t*	*p*	
YA vs MA	-0.07	0.02	654	-4.52	<.001	***
YA vs OA	-0.12	0.02	654	-7.52	<.001	***
YA vs Ad	-0.20	0.02	654	-10.68	<.001	***
MA vs OA	-0.05	0.02	654	-2.96	.019	***
MA vs Ad	-0.13	0.02	654	-6.73	<.001	***
OA vs Ad	-0.08	0.02	654	-4.30	<.001	***

Supplementary Table S2. Planned contrasts comparing face cognition accuracy overall between age groups. Abbreviations: YA – younger adolescents, MA – mid-adolescents, OA – older adolescents, Ad – adults, * $p < 0.05$, ** $p < 0.01$, *** $p < .001$

Contrast	Estimate	SE	*z*	*p*	
YA vs MA	-0.06	0.05	-1.36	1	
YA vs OA	-0.23	0.06	-4.04	<.001	***
YA vs Ad	-0.31	0.08	-3.69	.001	**
MA vs OA	-0.17	0.06	-2.82	.029	*
MA vs Ad	-0.24	0.08	-3.03	.015	*
OA vs Ad	-0.08	0.09	-0.87	1	

Supplementary Table S3. Planned contrasts comparing face cognition speed overall between age groups. Abbreviations: YA – younger adolescents, MA – mid-adolescents, OA – older adolescents, Ad – adults, * $p < 0.05$, ** $p < 0.01$, *** $p < .001$

**Contrast**	**Estimate**	**SE**	**df**	***t***	***p***	
YA vs MA	178.45	62.22	198.38	2.87	.028	*
YA vs OA	254.88	87.61	56.84	2.91	.031	*
YA vs Ad	559.34	165.43	10.68	3.38	.038	*
MA vs OA	76.43	75.49	109.11	1.01	1	
MA vs Ad	380.89	163.20	10.47	2.33	.244	
OA vs Ad	304.46	168.93	10.95	1.80	.594	

Supplementary Table S4. Planned contrasts comparing face perception accuracy between age groups and within processing component (identity, expression, gaze). Abbreviations: YA – younger adolescents, MA – mid-adolescents, OA – older adolescents, Ad – adults, * $p < 0.05$, ** $p < 0.01$, *** $p < .001$

**Contrast**	**Estimate**	**SE**	***z***	***p***	
identity: YA vs MA	-0.21	0.09	-2.32	.124	
identity: YA vs OA	-0.44	0.09	-4.80	<.001	***
identity: YA vs Ad	-0.58	0.11	-5.25	<.001	***
identity: MA vs OA	-0.23	0.09	-2.57	.061	
identity: MA vs Ad	-0.37	0.11	-3.45	.003	**
identity: OA vs Ad	-0.14	0.11	-1.34	1	
expression: YA vs MA	-0.06	0.09	-0.70	1	
expression: YA vs OA	-0.25	0.09	-2.69	.044	*
expression: YA vs Ad	-0.27	0.11	-2.43	.091	
expression: MA vs OA	-0.18	0.09	-2.03	.252	
expression: MA vs Ad	-0.21	0.11	-1.90	.341	
expression: OA vs Ad	-0.02	0.11	-0.21	1	
gaze: YA vs MA	-0.18	0.10	-1.86	.375	
gaze: YA vs OA	-0.12	0.10	-1.28	1	
gaze: YA vs Ad	-0.29	0.12	-2.43	.091	
gaze: MA vs OA	0.05	0.10	0.56	1	
gaze: MA vs Ad	-0.11	0.12	-0.94	1	
gaze: OA vs Ad	-0.17	0.12	-1.42	.930	

Supplementary Table S5. Planned contrasts comparing face perception accuracy between age groups and processing components: identity, expression and gaze. Abbreviations: YA – younger adolescents, MA – mid-adolescents, OA – older adolescents, Ad – adults, * $p < 0.05$, ** $p < 0.01$, *** $p < .001$

**Contrast**	**Estimate**	**SE**	***z***	***p***	
YA vs older: gaze vs expression	0.00	0.02	0.05	.963	
MA vs older: gaze vs expression	-0.06	0.04	-1.56	.118	
OA vs older: gaze vs expression	0.07	0.07	1.04	.299	
YA vs older: gaze vs identity	-0.05	0.02	-2.23	.026	*
MA vs older: gaze vs identity	-0.09	0.04	-2.58	.010	*
OA vs older: gaze vs identity	0.01	0.07	0.16	.872	

Supplementary Table S6. Planned contrasts comparing face perception speed between age groups and within processing component (identity, expression, gaze). Abbreviations: YA – younger adolescents, MA – mid-adolescents, OA – older adolescents, Ad – adults, * $p < 0.05$, ** $p < 0.01$, *** $p < .001$

**Contrast**	**Estimate**	**SE**	**df**	***t***	***p***	
identity: YA vs MA	143.60	46.84	436.90	3.07	.014	*
identity: YA vs OA	171.27	59.01	79.09	2.90	.029	*
identity: YA vs Ad	457.45	98.16	11.72	4.66	.004	**
identity: MA vs OA	27.67	53.58	168.29	0.52	1	
identity: MA vs Ad	313.85	97.02	11.61	3.24	.045	*
identity: OA vs Ad	286.18	100.29	11.86	2.85	.088	
expression: YA vs MA	173.28	46.82	437.05	3.70	.002	**
expression: YA vs OA	222.55	58.94	78.41	3.78	.002	**
expression: YA vs Ad	444.65	98.13	11.71	4.53	.004	**
expression: MA vs OA	49.26	53.50	168.92	0.92	1	
expression: MA vs Ad	271.36	97.03	11.62	2.80	.010	*
expression: OA vs Ad	222.10	100.27	11.85	2.22	.283	
gaze: YA vs MA	187.51	46.65	430.21	4.02	<.001	***
gaze: YA vs OA	185.65	58.93	78.57	3.15	.014	*
gaze: YA vs Ad	365.87	98.13	11.71	3.73	.018	*
gaze: MA vs OA	-1.86	53.36	167.51	-0.04	1	
gaze: MA vs Ad	178.36	96.97	11.59	1.84	.550	
gaze: OA vs Ad	180.22	100.27	11.85	1.80	.587	

Supplementary Table S7. Planned contrasts comparing face perception speed between age groups and processing components: identity, expression and gaze. Abbreviations: YA – younger adolescents, MA – mid-adolescents, OA – older adolescents, Ad – adults, * $p < 0.05$, ** $p < 0.01$, *** $p < .001$

**Contrast**	**Estimate**	**SE**	**df**	***t***	***p***
YA vs older: gaze vs expression	8.45	10.04	1291.80	0.84	.400
MA vs older: gaze vs expression	24.02	14.59	1293.40	1.65	.100
OA vs older: gaze vs expression	20.94	27.75	1289.20	0.76	.451
YA vs older: gaze vs identity	2.77	10.06	1293.00	0.28	.783
MA vs older: gaze vs identity	27.50	14.58	1292.90	1.89	.060
OA vs older: gaze vs identity	52.98	27.79	1290.20	1.91	.057

Supplementary Table S8. Nagelkerke's pseudo-R^2 (R_N^2) for the models used.

**Model predicting**	R_N^2
Face cognition accuracy	0.64
Face cognition speed	0.90
Face perception accuracy	0.49
Face perception speed	0.34
